# Warming Houses

**Published:** 1858-11

**Authors:** 


### WARMING HOUSES.

INDIFFERENCE, or motives of convenience, or false economy, are leading to the very general use of furnaces, which bear no proportion to the cheery comfort and healthfulness of the old-fashioned fire-place, and-irons, and solid hickory, ash, or sugar-tree, of delightful days departed, never to return. Most persons have found out, that furnaces do not keep a good-sized building comfortably warm when the thermometer is ten degrees or less above zero. In addition, the dry heat and gasy emanations from furnaces, are serious objections to their use. Several patterns claim to give a heat almost perfectly free from gas. BARTLETT'S furnace is the latest candidate for popular favor. We are trying it now, and in December, may be able to give an answer. Some of our intelligent and prominent citizens are highly pleased with it, after two seasons' trial. But we must speak from our own observation. We will tell only what it does for our house, and not say what it will do for others. As far as scientific principles are concerned, it is more nearly constructed in accordance with them than any furnace hitherto proposed. It is claimed, that less than three tons of coal will adequately heat a common three-story and basement dwelling. If it does this, and gives a heat free from gas, its inventor is a public benefactor, and, we trust, will be adequately rewarded.

As to the saving qualities of this furnace, a fair trial can only be made by purchasing a good article of coal. The difference between TRUSLOW'S coal, of Wall street, and what is advertised at four, five, or ten dimes below the ruling price, is sometimes as high as fifty per cent. We do not now remember a single article of household use, in which greater impositions are practiced, than in the coal we burn. Both coal and wood should be purchased from men whom we personally know to be correct in their dealings.

Last Winter, we threw our furnace out of doors, and used none, to the very great comfort and healthfulness of a rising family. If the pattern above is perfectly free from gas, we will use it, only on rare occasions of company, or great cold, and then merely for the halls, to take off the chill or dampness, making them no warmer than forty-five degrees.

